# Effect of an Enriched Protein Drink on Muscle Mass and Glycemic Control during Combined Lifestyle Intervention in Older Adults with Obesity and Type 2 Diabetes: A Double-Blind RCT

**DOI:** 10.3390/nu13010064

**Published:** 2020-12-28

**Authors:** Robert G. Memelink, Wilrike J. Pasman, Anke Bongers, Anita Tump, Annemieke van Ginkel, Wim Tromp, Suzan Wopereis, Sjors Verlaan, Johan de Vogel-van den Bosch, Peter J. M. Weijs

**Affiliations:** 1 Department of Nutrition and Dietetics, Faculty of Sports and Nutrition, Center of Expertise Urban Vitality, Amsterdam University of Applied Sciences, 1067 SM Amsterdam, The Netherlands; p.j.m.weijs@hva.nl; 2 Netherlands Organisation for Applied Scientific Research (TNO), 3704 HE Zeist, The Netherlands; wilrike.pasman@tno.nl (W.J.P.); suzan.wopereis@tno.nl (S.W.); 3 Danone Nutricia Research, Specialized Nutrition, 3584 CT Utrecht, The Netherlands; anke.bongers@nutricia.com (A.B.); johan.devogel@nutricia.com (J.d.V.-v.d.B.); 4 Vialente-Diëtheek, 3447 GW Woerden, The Netherlands; a.tump@dietheek.nl (A.T.); a.vanginkel@dietheek.nl (A.v.G.); 5 Tromp Medical, 1901 ND Castricum, The Netherlands; wtromp@trompmedical.com; 6 Department of Rehabilitation Medicine, Amsterdam University Medical Centers, Vrije Universiteit, 1081 HV Amsterdam, The Netherlands; g.verlaan@amsterdamumc.nl; 7 Department of Nutrition and Dietetics, Amsterdam University Medical Centers, Vrije Universiteit, 1081 HV Amsterdam, The Netherlands

**Keywords:** overweight, weight loss, diabetes, elderly, protein, lean mass, insulin sensitivity

## Abstract

Background: Weight loss is key to treatment of older adults with obesity and type 2 diabetes, but also a risk for muscle mass loss. This study investigated whether a whey protein drink enriched with leucine and vitamin D could preserve muscle mass and improve glycemic control during combined lifestyle intervention in this population. Methods: 123 older adults with obesity and type 2 diabetes were randomized into a 13-week lifestyle intervention with dietary advice and exercise, receiving either the enriched protein drink (test) or an isocaloric control (control). Muscle mass was assessed with dual-energy X-ray absorptiometry and glycemic control by oral glucose tolerance test. Statistical analyses were performed using a linear mixed model. Results: There was a nonsignificant increase in leg muscle mass (+0.28 kg; 95% CI, −0.01 to 0.56) and a significant increase in appendicular muscle mass (+0.36 kg; 95% CI, 0.005 to 0.71) and total lean mass (+0.92 kg; 95% CI, 0.19 to 1.65) in test vs. control. Insulin sensitivity (Matsuda index) also increased in test vs. control (+0.52; 95% CI, 0.07 to 0.97). Conclusions: Use of an enriched protein drink during combined lifestyle intervention shows beneficial effects on muscle mass and glycemic control in older adults with obesity and type 2 diabetes.

## 1. Introduction

The presence of type 2 diabetes in older adults with obesity accelerates the loss of muscle mass with ageing [1]. To reduce the disease burden of type 2 diabetes in people with obesity, the first therapy of choice is weight loss. A disadvantage of most weight loss interventions, however, is a decline in muscle mass, which comprises up to one third of total weight lost [2]. Preserving muscle mass during weight loss in this population is highly important, because skeletal muscle is strongly associated with physical performance outcomes in older adults [3], and is responsible for more than 75% of insulin-mediated glucose uptake [4].

Resistance exercise has been shown to be a promising strategy to overcome the loss of muscle mass during weight loss [5]. It reduces muscle mass loss during weight loss in frail older adults with obesity [6] and also improves insulin sensitivity of the muscles in older adults with obesity and type 2 diabetes [7]. Increasing protein intake is another promising strategy, especially when combined with resistance exercise. The combination of an energy-restricted, high-protein diet and resistance training (3 times/week) showed greater weight loss and fat mass loss than either intervention alone in type 2 diabetes patients with overweight or obesity [8]. However, the overall reduction in fat-free mass did not differ between groups.

Currently, there is no clear evidence on the effect of dietary protein supplementation along with exercise on muscle mass preservation and glycemic control during weight loss in older adults with obesity and type 2 diabetes. A recent study in older adults with obesity showed that the combination of a whey protein drink enriched with leucine and vitamin D with resistance exercise resulted in preservation of muscle mass during weight loss [9]. Especially the branched-chain amino acid leucine is known for stimulating muscle protein synthesis [10]. Leucine may enhance muscle protein synthesis through its insulinotropic effect, increasing amino acid availability for muscle protein synthesis [11]. Vitamin D supplementation has a positive impact on muscle strength in older adults [12], and improves insulin sensitivity in older adults with impaired insulin sensitivity [13].

We aim to assess whether a whey protein drink enriched with leucine and vitamin D (test drink) supports muscle mass preservation and improves glycemic control as part of a lifestyle intervention with hypocaloric diet, resistance exercise, and high-intensity interval training (HIIT) in older adults with obesity and type 2 diabetes. We hypothesize that the test drink has a beneficial effect on muscle mass and glycemic control compared to an isocaloric control drink in these older adults with obesity and type 2 diabetes.

## 2. Materials and Methods

### 2.1. Subjects

Older adults (≥55 years) with obesity and type 2 (pre-)diabetes were recruited from the Dutch population through local flyers and regional advertisements. Obesity was defined as having a BMI >30 kg/m^2^, or a BMI >27 kg/m^2^ with waist circumference >88 cm (women) or >102 cm (men). Type 2 diabetes was defined as using diabetes medication. Pre-diabetes was defined as having a blood hemoglobin A1c level (HbA1c) ≥43 mmol/mL. Out of 15 pre-diabetes participants included, 11 were classified as having diabetes type 2 based on baseline measurements of HbA1c (>47 mmol/L), fasting plasma glucose (>7.0 mmol/L), or 2 h plasma glucose (>11.1 mmol/L) during oral glucose tolerance test (OGTT). Potential participants were excluded if they suffered from any malignant disease during the last five years, if they used insulin, if they had renal or hepatic disease, if they followed any specific diet within three months before screening, if participation in the exercise program was considered unsafe based on exercise ECG and anamnesis by a sports physician, or if there was uncertainty about their ability to fully comply with the study protocol. A full description of the eligibility criteria is available in the Netherlands Trial Register (www.trialregister.nl), where the study was registered under number NL4357. The study was approved by the Medical Ethics Committee Assen, The Netherlands (NL46790.056.14), and was performed in accordance with the Helsinki Declaration of 1975 as revised in 1983. The study team obtained written informed consent from all subjects. The study took place from September 2014 until January 2017 at the Amsterdam University of Applied Sciences, Amsterdam, The Netherlands. Subjects visited the Amsterdam Nutritional Assessment Center and the adjacent fitness center for all study related activities, except for the glycemic control measurements, which were performed at the Amsterdam University Medical Centers, location VUmc, Amsterdam, The Netherlands.

### 2.2. Design and Randomization Procedures

We performed a 13-week randomized, controlled, double-blind, parallel group trial. Randomization was stratified by sex and use of sulfonylurea (SU) derivatives at study start (yes/no) to prevent any effect on muscle mass of an uneven distribution over the study groups. SU-derivatives stimulate insulin production in the beta cells in the pancreas [14], and insulin, in its turn, increases the rate of protein synthesis and decreases the rate of protein breakdown in muscle [15].

The randomization list was generated by an independent statistician who was not involved in conducting the study. According to the order on the randomization list, study staff randomly allocated eligible subjects (1:1, with block size 10) to receive a whey protein drink enriched with leucine and vitamin D (test group) or an isocaloric control drink (control group) during the intervention. Muscle mass and other parameters of body composition, parameters of glycemic control, muscle strength, and physical performance were assessed at baseline and after 13 weeks of intervention. Body weight, BMI, and waist circumference were measured at baseline and after 7 and 13 weeks of intervention. Subjects and all study staff were blinded to the study products.

### 2.3. Hypocaloric Diet

All subjects were instructed to adhere to a hypocaloric diet of 600 kcal below estimated energy needs according to the Dutch guideline for treatment of obesity [16], including the caloric content of the study products. Energy needs were based on measured resting energy expenditure by indirect calorimetry (Vmax Encore n29; CareFusion) multiplied by the physical activity level (PAL) estimated with an accelerometer (PAM AM200; PAM BV). Resting energy expenditure (REE) was measured after an overnight fast. Subjects reported to our lab on the morning of the measurement, travelling by car or bus to prevent increased energy expenditure. After a 30-min acclimatization in the lab, REE was measured for 30 min and the first 5 min of the measurement were neglected. A steady state period (coefficient of variation <10%) of at least 10 min was used for calculation of REE. The accelerometer was worn on subject’s belt or rim of their trousers or skirt, between the right hip and the belly button. Subjects wore the accelerometer for three consecutive days, during waking hours on two weekdays and one weekend day, at baseline and after 13 weeks of intervention.

Throughout the 13-week intervention, subjects followed six individual dietary counselling sessions and six nutrition and lifestyle group sessions. Dietary intake was assessed by a three-day food record at baseline and after 13 weeks of intervention, on the same three days as the accelerometer was worn. Food records were checked for completeness with participants during study visits and additional information was obtained about unclear items or amounts. Total energy and macronutrient intakes were calculated using the Dutch Food Composition Database, version 2013/4.0 [17].

### 2.4. Exercise Program 

All subjects participated in the exercise program, which was conducted three times per week in 1 h group sessions under supervision of a qualified personal trainer for a period of 13 weeks. The exercise program consisted of progressive resistance exercise and high intensity interval training (HIIT). Resistance exercise involved a selection of 10 exercises focusing on the large muscle groups in arms and legs. Subjects were familiarized to the exercises during the first week of intervention. After familiarization, exercise intensity started at 60% of subject’s one repetition maximum (1-RM) and progressed to 80% of 1-RM throughout the intervention. The number of sets increased from one to three according to individual progress. After two weeks of training, subjects started HIIT on a cycle ergometer at 70% of their maximal work capacity, determined using a steep ramp test [18]. Intervals of 30 s of high-intensity exercise were followed by 60 s of active rest. Exercise intensity progressed to 110% of maximal work capacity throughout the intervention. The number of intervals progressed from four to eight, according to individual progress [19]. Each training session was preceded by a 10-min warming up on a cycle ergometer, cross trainer, or treadmill. Further details on the exercise program are provided in Supplementary Document S1.

### 2.5. Study Products

Study products, in identical sachets and sequentially numbered corresponding with the randomization numbers, were provided by Danone Nutricia Research, Utrecht, The Netherlands. The test product was a whey protein drink enriched with leucine and vitamin D, containing 21 g leucine-enriched whey protein (3 g total leucine), 9 g carbohydrates, 3 g fat, 800 IU cholecalciferol (VITAMIN D_3_), and a mixture of vitamins, minerals, and fibers. The control product contained 25 g carbohydrates and 6 g fat. Both products were similar in taste and appearance, provided an energetic value of 150 kcal per serving, and were dissolved in 150 mL water just before consumption. A detailed description of the study products is given in Appendix A. Subjects were asked to consume 10 servings of the study product per week throughout the 13-week intervention period; one serving just before breakfast each day (7 times/week), and another serving immediately after each training session (3 times/week). Study products had to be consumed as a single bolus within 5–10 min. To verify adherence to intake of the study products, subjects were asked to record product intake in a daily diary, and serum calcidiol (nmol/L) was assessed. 

### 2.6. Measurement of Body Composition and Anthropometry

Body composition was measured with dual-energy X-ray absorptiometry (DXA; Hologic Discovery A; Hologic). To limit within-subject variation, DXA scans at both measurements were performed with subjects in fasting state, wearing underwear, and after a toilet visit. One single staff member performed analysis of all DXA scans to exclude between-assessor variability. Regional cut-points were set by the Hologic software and were adjusted in case the software failed to achieve the standard demarcations. An external blinded expert (A.S.) reviewed all interpretation issues related to the DXA scans. The primary outcome parameter leg muscle mass (kg) was defined as the sum of lean mass (without bone) of both legs. Appendicular muscle mass (kg; secondary outcome parameter) was defined as the sum of lean mass (without bone) of both arms and legs. Other parameters derived from DXA were total fat mass (kg; secondary outcome parameter), total lean mass (kg), and visceral adipose tissue area (VAT; cm^2^).

Body weight (secondary outcome parameter) was measured to the nearest 0.01 kg on a calibrated scale (Life Measurement). Height was measured to the nearest 0.1 cm using a wall-mounted stadiometer (De Grood DGI 250D; De Grood metaaltechniek). Body weight and height were used to calculate BMI (kg/m^2^). Waist circumference was measured to the nearest 0.1 cm using measuring tape.

### 2.7. Measurement of Glycemic Control

Glycemic control was assessed using a 2 h OGTT upon overnight fasting. Venous blood samples were taken from an indwelling catheter immediately before (t = 0) and 30, 60, 90, and 120 min after consumption of a 300 mL 75 g glucose solution (Added Pharma). Samples were analyzed for plasma glucose (mmol/L) and insulin (pmol/L). The fasting sample was analyzed for HbA1c (mmol/mol). Fasting plasma glucose, 2 h plasma glucose, and HbA1c were secondary outcome parameters. Homeostatic model assessment for insulin resistance (HOMA-IR) and Matsuda index were calculated from the OGTT, as measures for systemic insulin resistance [20] and insulin sensitivity [21], respectively:

(1) $$\mathrm{HOMA}\;\mathrm{IR}=\frac{\mathrm{fasting}\;\mathrm{insulin}\;\left(\frac{\mathrm{mU}}{L}\right)\times \mathrm{fasting}\;\mathrm{glucose}\;\left(\frac{\mathrm{mmol}}{L}\right)}{22.5}$$

(2) $$\mathrm{Matsuda}\;\mathrm{index}=\frac{10,000}{\sqrt{\left(\mathrm{fasting}\;\mathrm{insulin}\;\left(\frac{\mathrm{mU}}{L}\right)\times \mathrm{fasting}\;\mathrm{glucose}\;\left(\frac{\mathrm{mg}}{\mathrm{dL}}\right)\right)\times \left(\mathrm{mean}\;\mathrm{insulin}\;\left(\frac{\mathrm{mU}}{L}\right)\times \mathrm{mean}\;\mathrm{glucose}\;\left(\frac{\mathrm{mg}}{\mathrm{dL}}\right)\right)}}$$

Diabetes medication and any changes were recorded throughout the intervention. To prevent increased risk of hypoglycemic events during the intervention, subjects using SU derivatives were intensively monitored for glucose level during both training days and non-training days. If necessary in the opinion of the study physician, medication dose was adapted at the start or during intervention. 

### 2.8. Measurement of Muscle Strength and Power and Physical Performance

10-RM leg press strength (kg) was measured using a leg press machine (Technogym Selection; Technogym). Knee extension power (W) was measured using a leg extension machine (Technogym Selection; Technogym), with a linear encoder attached to the weight stack (Humac 360; CSMi) [22]. Knee extension power was defined as the maximum average power obtained during 5–7 knee extensions at maximal speed with increasing weights. Physical performance was assessed with the 400 m fast paced walk (m/s) [23], the short physical performance battery (SPPB) consisting of a balance test, a 4 m usual gait speed test (m/s), and a chair stand test (s) [24], and a steep ramp test performed on a cycle ergometer to determine peak oxygen uptake (VO_2_peak; L/min) (Quark RMR/CPET; Cosmed) [19].

### 2.9. Statistical Analysis

Sample size assumptions were based on the study of Verreijen et al. [9], evaluating the effect of the same test drink during intentional weight loss in older adults with obesity. A sample size of 44 per arm provided 80% power to detect an absolute difference of 0.92 kg leg muscle mass with an SD of 1.51 kg and *p* < 0.05 (2-sided). Assuming a dropout rate of 25%, 118 subjects were needed for the study. Because subjects were enrolled in five different clusters, we aimed to enroll approximately 24 subjects per cluster. Finally, 123 subjects were enrolled in the study.

Single data entry with 100% data monitoring for primary and secondary outcome parameters was performed and discrepancies were solved. Statistical analysis was based on the intention-to-treat (ITT) dataset, including all 123 randomized subjects. For body weight and body composition, statistical analysis was based on a modified ITT dataset, excluding 1 of the 123 randomized subjects after unblinding, who was identified as a statistically influential outlier for leg muscle mass (Supplementary Figure S1). This subject had reported oedema in the legs at baseline and started on a sodium-restricted diet during the study. The resulting loss of fluid (as reported by the subject) very likely influenced the assessments of body weight and body composition (DXA). 

Subject characteristics will be presented using descriptive statistics, and dietary intake will be compared between study groups using independent samples t-test. Between and within group differences in primary, secondary, and all other outcome variables were analyzed using a linear mixed model with a random effect for subjects and a fixed factor for study group, time, sex, use of SU derivatives at study start (yes/no), and time * study group interaction. Between group differences in study product compliance, adherence to the training program, and change in diabetes medication were analyzed using a Fisher’s Exact Test. A sensitivity analysis was performed by excluding the subjects with pre-diabetes, to evaluate whether their inclusion in the study affected the results of our main analysis.

SPSS version 24 statistics software (IBM Corporation) was used for all statistical analyses. Subject characteristics and dietary intake are presented as observed mean ± SD, or number with percentage. Outcome parameters are presented as estimated marginal mean (EMM) ± SE. Intervention effects are presented as EMM with 95% confidence interval (CI). Statistical significance was defined as a 2-tailed *p* < 0.05.

## 3. Results

### 3.1. Subjects, Safety, and Compliance to Study Product Intake and Exercise Program

**Figure 1:**
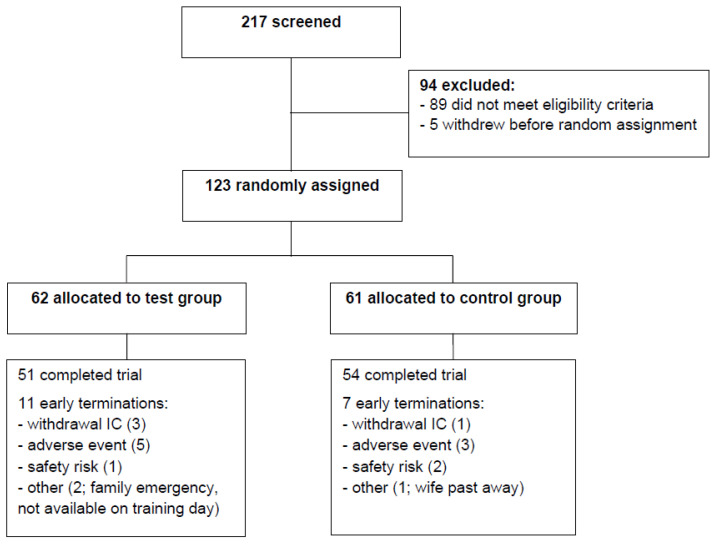
Flow chart of the PROBE study, a double-blind, randomized, controlled trial on the effect of a protein drink enriched with leucine and vitamin D on lean mass and glycemic control during a combined lifestyle intervention in older adults with obesity and type 2 diabetes. IC: informed consent.

Out of the 123 subjects enrolled, 18 dropped out of the study because of personal reasons (*n* = 6), increased risk of hypoglycemia (*n* = 3), or adverse events (*n* = 9, unrelated to the study products). The number of subjects screened, excluded, enrolled, and randomly allocated is shown in Figure 1.

There were no clinically relevant effects on vital signs or blood parameters for liver and kidney function. Overall, there were 17 adverse events (AEs) related to the study products, without significant differences between study groups (six events in the test group; 11 events in the control group). AE severity was mostly rated as mild, and the subjects did not require treatment. Four serious adverse events (SAEs) were reported, unrelated to the intervention. In the test group, one subject was diagnosed with prostate cancer with metastasis, and another subject had a light myocardial infarction. In the control group, one subject was diagnosed with breast cancer, and another subject had severe pneumonia. 

There were no relevant differences in subject characteristics between study groups (Table 1). Study product compliance did not differ between groups: 90% of subjects (test group) compared to 97% (control group) consumed at least seven units of study product per week (*p* = 0.283). Adherence to the exercise program did not differ between groups: 82% of subjects (test group) compared to 89% (control group) attended at least two out of three exercise sessions per week (*p* = 0.467).

### 3.2. Body Weight, Body Composition, and Anthropometry 

During the combined lifestyle intervention, we observed a significant reduction in body weight (−2.6 ± 0.3 kg; *p* < 0.001), without statistically significant differences between groups. BMI, fat mass, waist circumference, and VAT also decreased over time without significant differences between groups (Table 2).

**Figure 2:**
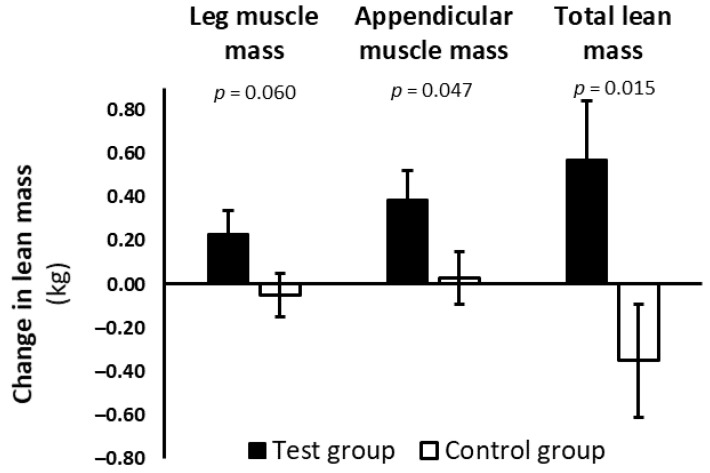
Change in leg muscle mass, appendicular muscle mass, and total lean mass in test group and control group after 13 weeks of combined lifestyle intervention in the PROBE study. Data are presented as estimated marginal mean ± SE. Analysis was based on a modified ITT population, excluding a statistically influential outlier for LMM (see methods). *p*-value indicates significance level of the estimate of group difference. ■ Test group; ☐ Control group.

Leg muscle mass (+0.23 ± 0.11; *p* = 0.030), appendicular muscle mass (+0.39 ± 0.13; *p* = 0.003), and total lean mass (+0.57 ± 0.27; *p* = 0.034) increased in the test group only. The control group showed neither gain nor loss in leg muscle mass (−0.05 ± 0.10; *p* = 0.655), appendicular muscle mass (+0.03 ± 0.12; *p* = 0.795), and total lean mass (−0.35 ± 0.26; *p* = 0.179). In test compared to control, there was a statistically nonsignificant increase in leg muscle mass (+0.28 kg; 95% CI, −0.01 to 0.56; *p* = 0.060), and a statistically significant increase in appendicular muscle mass (+0.36 kg; 95% CI, 0.005 to 0.71; *p* = 0.047) and total lean mass (+0.92 kg; 95% CI, 0.19 to 1.65; *p* = 0.015) (Figure 2). Full ITT analysis results for body composition are shown in Supplementary Table S1. Sensitivity analysis, excluding the subjects with pre-diabetes, demonstrated a statistically significant increase in leg muscle mass (+0.32 kg; 95% CI, 0.03 to 0.62; *p* = 0.030), appendicular muscle mass (+0.42 kg; 95% CI, 0.06 to 0.78; *p* = 0.021), and total lean mass (+1.03 kg; 95% CI, 0.29 to 1.77; *p* = 0.007), in test compared to control (Supplementary Table S2).

### 3.3. Glycemic Control

During the combined lifestyle intervention we observed significant reductions in fasting plasma glucose (−0.67 ± 0.16 mmol/L; *p* < 0.001), 2 h plasma glucose (−1.11 ± 0.26 mmol/L; *p* < 0.001), and HbA1c (−5.0 ± 0.8 mmol/mol; *p* < 0.001), without differences between groups. Fasting plasma insulin, HOMA-IR, and Matsuda index improved over time in the test group only (fasting insulin: −20.1 ± 6.5 pmol/L; *p* = 0.003, HOMA-IR: −1.40 ± 0.41; *p* = 0.001, Matsuda index: +0.52 ± 0.16; *p* = 0.002) and were significantly different compared to the control group (Table 2). Sensitivity analysis, excluding the subjects with pre-diabetes, confirmed these findings (Supplementary Table S2).

In 30 out of 108 diabetes medication users (28%), diabetes medication was reduced (14 at the start of intervention, 15 during intervention, one both at start and during intervention). None of the subjects had an increase in diabetes medication. There was no difference in the number of subjects with reduced diabetes medication between the test group (*n* = 14) and control group (*n* = 16) (*p* = 0.924).

### 3.4. Muscle Strength, Muscle Power, and Physical Performance

During the combined lifestyle intervention we observed significant improvements in 10-RM leg press strength, knee extension power, 400-m walk speed, chair stand time, and VO_2_peak, without statistical differences between groups (Table 2). Usual gait speed and PAL did not significantly change over time and was not different between groups.

### 3.5. Dietary Intake

Baseline energy needs were not significantly different between the test (2180 ± 361 kcal/d) and the control group (2203 ± 446 kcal/d) (*p* = 0.777). According to the self-reported three-day food record at week 13, energy intake during intervention (including the study product) was not significantly different between the test and the control group (Table 3). Protein intake at week 13 was higher in the test group compared to the control group (+0.33 g⋅kg body weight^−1^⋅d^−1^; *p* < 0.001), reflecting the difference in protein content between the study products. Consequently, the contribution of both carbohydrates and fat to the total dietary energy intake at week 13 was lower in the test group than in the control group (*p* < 0.001, resp. *p* = 0.037). Mean serum calcidiol level changed from 63.8 to 82.5 nmol/L in the test group, and from 61.2 to 57.9 nmol/L in the control group. This was significantly different between the groups and reflects the difference in vitamin D content between the study products.

## 4. Discussion

This 13-week combined lifestyle intervention with a whey protein drink enriched with leucine and vitamin D in older adults with obesity and type 2 diabetes showed a beneficial effect on preservation of muscle mass during weight loss in the test group compared to the control group. During lifestyle intervention, a reduction in fasting plasma glucose, 2 h plasma glucose, and HbA1c was observed in both groups, without between group differences. The combination of the lifestyle intervention with the test drink reduced fasting insulin and improved the Matsuda index compared to the control drink. 

Although the amount of body weight lost during intervention (2.6 kg) was less than expected, it is more or less comparable to other weight loss studies that use dietary advice and or coaching [9,25,26,27,28]. A majority of the subjects did not reach the energy restriction target of 600 kcal/day, and subjects probably underreported their energy intake, which is common in patients with obesity [29]. Recent lifestyle interventions involving exercise and a hypocaloric diet in (older) adults with obesity and type 2 diabetes also showed significant reductions in body weight and body fat, but failed to show preservation of muscle mass during weight loss [8,30]. The large prospective Look AHEAD study showed that intensive coaching on reducing caloric intake and increasing physical activity (brisk walking or similar aerobic activity and unsupervised at-home exercise) is not enough to prevent aging-related loss of leg muscle mass [30]. Wycherley et al. [8] evaluated the separate and combined effects of 16 weeks of energy-restricted high-protein diet and resistance exercise on weight loss and body composition in patients with overweight/obesity and type 2 diabetes. Their approach with partially provided diets resulted in approximately 10 kg loss of body weight, of which approximately 2 kg was fat-free mass. There was no effect of the high protein diet and/or resistance exercise on the preservation of fat-free mass. In frail older adults with obesity, Villareal et al. [25] evaluated the effects of both diet and exercise in a one-year RCT. After six months, loss of lean body mass was 24% of total weight lost in the combined diet + exercise group. The reasons for absence of muscle preservation in the above mentioned studies could be sub-optimal quantity or quality of dietary protein intake [31]. Previously, Verreijen et al. [9] showed preservation of appendicular muscle mass using a whey protein drink enriched with leucine and vitamin D during a combined lifestyle intervention in older adults with obesity. In the current study, we confirmed the beneficial effect of the whey protein drink enriched with leucine and vitamin D with an increase in appendicular muscle mass during significant weight loss. Remarkably, we could not reproduce the loss of leg or appendicular muscle mass in the control group during the combined lifestyle intervention, despite comparable dietary intake in both studies. This might be due to a difference in health status (type 2 diabetes), or more likely a difference in training protocol. The addition of HIIT training on top of resistance exercise may have had additional anabolic effects. HIIT augments skeletal muscle fiber recruitment, which may have resulted in greater improvements in microvascular function [32]. In type 2 diabetes, improvement of microvascular perfusion has been suggested as new potential target to prevent or decrease muscle loss [33]. Compared to other training regimens, HIIT leads to expression of the largest number of genes in mitochondrial, muscle growth, and insulin signaling pathways in older adults [34]. Thus, the addition of HIIT to the resistance exercise program, compared to the training as conducted by Verreijen et al. [9], might explain the preservation of appendicular muscle mass in the control group. Contrary to the preservation of appendicular muscle mass, a decreased total lean mass was observed in the control group, suggesting that preservation occurred in the most intensively trained (i.e., appendicular) muscles only. By providing additional protein of high quality, this study showed that it is possible to preserve muscle mass during weight loss in older adults with obesity and type 2 diabetes. Validation of this finding in an independent study is important to confirm our observation. 

Overall, the 13-week combined lifestyle intervention led to clinically relevant reductions in fasting plasma glucose, 2 h plasma glucose, and HbA1c. This has been shown earlier in weight loss interventions in (older) adults with obesity or type 2 diabetes [35,36]. While both resistance exercise and HIIT are beneficial for insulin sensitivity in patients with (or at risk of) type 2 diabetes [7,37], the whey protein drink enriched with leucine and vitamin D in the current study appeared to have an additional beneficial effect on glycemic control, reflected by a lower fasting plasma insulin concentration and improved HOMA-IR and Matsuda index as compared to control. These differences between the groups may be explained by the study product composition (protein, micronutrient, and carbohydrate content), improved insulin sensitivity, and/or insulin uptake capacity by e.g., increased muscle mass [38]. Increased intake of high quality dietary protein has been reported to improve insulin sensitivity in older adults with type 2 diabetes who have a normal weight or are overweight [39]. One of the characteristics of the test drink is the high level of leucine, which might have played a role in the improved glycemic control, due to its strong insulinotropic characteristics [11]. Vitamin D from the test product may have played a role as well. Talaei et al. [40] showed significant improvements in insulin resistance in patients with type 2 diabetes after treatment with a six times higher dose of vitamin D. However, this potential effect of vitamin D remains subject of future studies. The observed reductions in fasting glucose and HbA1c during the combined lifestyle intervention in our study are promising findings for clinical practice, especially because these reductions are accompanied by reductions in diabetes medication.

Muscle strength, muscle power, and physical performance are important to sustain independent mobility and healthy lifestyle during ageing [41]. Overall, muscle strength, muscle power, and physical performance improved in both groups, at levels comparable to those reported by Villareal et al. [22], but we did not observe differences between groups. These results are not in line with a recent meta-analysis of Liao et al. [42] who showed that overweight or obese older adults had substantially greater leg strength gain, in addition to greater lean mass gain, when protein supplementation was combined with resistance exercise, compared to resistance exercise alone. It is, however, unclear whether participants were on a hypocaloric diet in the selected studies in this meta-analysis. The absence of muscle mass loss in the control group in our study may also have contributed to the absence of difference in strength, power, and performance between the groups.

### Strengths and Limitations

The current results should be interpreted with strengths and limitations of the study. The double-blind randomized controlled design is a major strength of the study. Additionally, subjects showed a high level of training compliance and study product compliance. Though results on body composition were based on modified ITT analysis, full ITT analysis still points towards effectiveness of the whey protein drink enriched with leucine and vitamin D in preserving lean mass during combined lifestyle intervention (Supplementary Table S1). The inclusion of subjects with pre-diabetes could be seen as a limitation of the study, but this did not bias our results, as was demonstrated by a sensitivity analysis excluding these four subjects. The sensitivity analysis confirmed the findings on body composition and glycemic control from our main analyses (Supplementary Table S2).

## 5. Conclusions

The use of a whey protein drink enriched with leucine and vitamin D during a combined lifestyle intervention shows beneficial effects on muscle mass and glycemic control in older adults with obesity and type 2 diabetes.

## Figures and Tables

**Table 1:** Baseline characteristics of the PROBE study subjects, by treatment.

Characteristic	*n*	Test Group	*n*	Control Group
Male sex, *n* (%)	62	42 (68)	61	38 (62)
Origin, % Caucasian	62	86	61	85
Age, years	62	66.8 ± 6.0	61	65.8 ± 6.4
Body weight, kg	62	98.17 ± 14.99	61	100.07 ± 15.59
BMI, kg/m^2^	62	32.8 ± 4.4	61	33.5 ± 4.6
Waist circumference, cm	61	114.1 ± 9.4	60	115.9 ± 10.7
Fat mass, %	61	33.5 ± 7.0	61	34.3 ± 6.0
Leg muscle mass, kg	61	19.59 ± 3.69	61	19.79 ± 3.45
Appendicular muscle mass, kg	59	26.47 ± 5.29	61	26.85 ± 5.10
Total lean mass, kg	60	63.17 ± 10.66	61	64.04 ± 10.96
Skeletal muscle mass index, kg/m^2^	59	8.83 ± 1.21	61	8.93 ± 1.10
Duration of diabetes, months	58	94 ± 83	56	78 ± 57
Use of diabetes medication, *n* (%)	62	53 (86)	61	55 (90)
Use of SU derivatives, *n* (%)		21 (34)		23 (38)
Use of metformin, *n* (%)		49 (79)		52 (85)
No medication, *n* (%)		9 (15)		6 (10)
Fasting glucose, mmol/L	57	8.25 ± 1.76	58	8.24 ± 1.90
HbA1c, mmol/mol	60	51.08 ± 9.66	58	52.95 ± 10.86
Fasting insulin, pmol/L	60	116.1 ± 73.2	59	102.0 ± 41.5
Serum calcidiol, nmol/L	57	63.0 ± 28.1	58	60.4 ± 18.0
Handgrip strength, kg	60	36.3 ± 10.8	60	36.5 ± 10.3
400-m walk speed, m/s	61	1.40 ± 0.22	58	1.49 ± 0.23
Usual gait speed, m/s	62	1.12 ± 0.22	60	1.18 ± 0.21
Chair stand, s	62	11.9 ± 2.5	60	11.5 ± 2.5
PAL	52	1.19 ± 0.07	57	1.20 ± 0.09
Current smoker, *n* (%)	62	7 (11)	61	6 (10)
Alcohol user, *n* (%)	62	46 (74)	61	38 (62)

Data are presented as mean ± SD or as number (percentage). BMI, body mass index; SU, sulfonylurea; HbA1c, haemoglobin A1c; PAL, physical activity level.

**Table 2:** Outcome measures for test and control group with intervention effect.

	Test Group	Control Group	Intervention Effect
			Beta (95% CI) ^a^
Body weight ^b^, kg			
Baseline (*n*)	96.11 ± 1.97 (61)	98.94 ± 1.91 (61)	
Change (*n*)	−2.23 ± 0.41 (50)	−2.92 ± 0.39 (54)	0.69 (−0.44 to 1.82)
*p* value	<0.001	<0.001	0.226
BMI ^b^, kg/m^2^			
Baseline (*n*)	32.9 ± 0.6 (61)	33.7 ± 0.6 (61)	
Change (*n*)	−0.7 ± 0.1 (50)	−1.0 ± 0.1 (54)	0.2 (−0.2 to 0.6)
*p* value	<0.001	<0.001	0.252
Leg muscle mass ^b^, kg			
Baseline (*n*)	18.69 ± 0.36 (60)	19.19 ± 0.34 (61)	
Change (*n*)	0.23 ± 0.11 (49)	−0.05 ± 0.10 (54)	0.28 (−0.01 to 0.56)
*p* value	0.030	0.655	0.060
Appendicular muscle mass ^b^, kg			
Baseline (*n*)	25.22 ± 0.48 (58)	25.90 ± 0.46 (61)	
Change (*n*)	0.39 ± 0.13 (47)	0.03 ± 0.12 (51)	0.36 (0.005 to 0.71)
*p* value	0.003	0.795	0.047
Total lean mass ^b^, kg			
Baseline (*n*)	60.44 ± 1.01 (59)	62.11 ± 0.98 (61)	
Change (*n*)	0.57 ± 0.27 (48)	−0.35 ± 0.26 (52)	0.92 (0.19 to 1.65)
*p* value	0.034	0.179	0.015
Fat mass ^b^, kg			
Baseline (*n*)	34.30 ± 1.17 (60)	35.24 ± 1.13 (61)	
Change (*n*)	−2.63 ± 0.33 (49)	−2.60 ± 0.32 (52)	−0.03 (−0.96 to 0.89)
*p* value	<0.001	<0.001	0.941
Waist circumference ^b^, cm			
Baseline (*n*)	113.1 ± 1.4 (60)	115.1 ± 1.3 (60)	
Change (*n*)	−3.4 ± 0.5 (49)	−3.7 ± 0.5 (52)	0.2 (−1.2 to 1.7)
*p* value	<0.001	<0.001	0.729
VAT ^b^, cm^2^			
Baseline (*n*)	177.2 ± 7.1 (61)	181.1 ± 6.8 (61)	
Change (*n*)	−18.9 ± 3.9 (50)	−17.3 ± 3.8 (54)	−1.6 (−12.5 to 9.3)
*p* value	<0.001	<0.001	0.772
Fasting plasma glucose, mmol/L			
Baseline (*n*)	8.38 ± 0.23 (57)	8.34 ± 0.23 (58)	
Change (*n*)	−0.68 ± 0.23 (47)	−0.66 ± 0.23 (50)	−0.03 (−0.67 to 0.61)
*p* value	0.004	0.004	0.936
2h plasma glucose, mmol/L			
Baseline (*n*)	15.82 ± 0.45 (57)	15.61 ± 0.44 (57)	
Change (*n*)	−0.93 ± 0.37 (47)	−1.29 ± 0.37 (47)	0.37 (−0.66 to 1.40)
*p* value	0.013	0.001	0.477
HbA1c, mmol/mol			
Baseline (*n*)	52.3 ± 1.2 (60)	53.8 ± 1.2 (58)	
Change (*n*)	−4.4 ± 1.1 (49)	−5.7 ± 1.1 (51)	1.3 (−1.7 to 4.4)
*p* value	<0.001	<0.001	0.390
Fasting plasma insulin, pmol/L			
Baseline (*n*)	119.4 ± 7.8 (60)	104.8 ± 7.6 (59)	
Change (*n*)	−20.1 ± 6.5 (48)	9.4 ± 6.4 (50)	−29.5 (−47.6 to −11.4)
*p* value	0.003	0.147	0.002
HOMA-IR			
Baseline (*n*)	6.32 ± 0.44 (57)	5.52 ± 0.44 (57)	
Change (*n*)	−1.40 ± 0.41 (46)	0.12 ± 0.40 (49)	−1.52 (−2.65 to −0.39)
*p* value	0.001	0.769	0.009
Matsuda index			
Baseline (*n*)	2.15 ± 0.18 (55)	2.19 ± 0.17 (55)	
Change (*n*)	0.52 ± 0.16 (43)	0.00 ± 0.16 (44)	0.52 (0.07 to 0.97)
*p* value	0.002	0.980	0.023
Serum calcidiol, nmol/L			
Baseline (*n*)	63.8 ± 2.9 (57)	61.2 ± 2.8 (58)	
Change (*n*)	18.7 ± 2.8 (45)	−3.3 ± 2.7 (48)	22.0 (14.2 to 29.7)
*p* value	<0.001	0.236	<0.001
10-RM leg press, kg			
Baseline (*n*)	125 ± 8 (55)	121 ± 8 (54)	
Change (*n*)	49 ± 7 (36)	56 ± 6 (41)	−7 (−26 to 12)
*p* value	<0.001	<0.001	0.462
Knee extension power, Watt			
Baseline (*n*)	334 ± 17 (49)	345 ± 16 (53)	
Change (*n*)	30 ± 8 (34)	35 ± 8 (39)	−5 (−27 to 17)
*p* value	<0.001	<0.001	0.652
400-m walk speed, m/s			
Baseline (*n*)	1.37 ± 0.03 (61)	1.46 ± 0.03 (58)	
Change (*n*)	0.07 ± 0.02 (48)	0.04 ± 0.02 (51)	0.04 (−0.01 to 0.09)
*p* value	<0.001	0.044	0.166
Usual gait speed, m/s			
Baseline (*n*)	1.11 ± 0.03 (62)	1.17 ± 0.03 (60)	
Change (*n*)	0.02 ± 0.03 (50)	−0.03 ± 0.03 (53)	0.04 (−0.03 to 0.12)
*p* value	0.594	0.325	0.286
Chair stand, s			
Baseline (*n*)	12.1 ± 0.3 (62)	11.7 ± 0.3 (58)	
Change (*n*)	−1.4 ± 0.3 (50)	−1.2 ± 0.3 (50)	−0.2 (−0.9 to 0.6)
*p* value	<0.001	<0.001	0.677
VO_2_peak, l/min			
Baseline (*n*)	1.60 ± 0.05 (61)	1.76 ± 0.05 (60)	
Change (*n*)	0.13 ± 0.04 (42)	0.11 ± 0.03 (48)	0.02 (−0.08 to 0.12)
*p* value	0.001	0.002	0.665
PAL			
Baseline (*n*)	1.18 ± 0.01 (52)	1.19 ± 0.01 (57)	
Change (*n*)	0.01 ± 0.01 (41)	0.00 ± 0.01 (45)	0.01 (−0.02 to 0.04)
*p* value	0.335	0.833	0.580

Data are presented as estimated marginal mean ± SE. CI, confidence interval; BMI, body mass index; VAT, visceral adipose tissue; HbA1c, haemoglobin A1c; HOMA-IR, homeostatic model assessment for insulin resistance; 10-RM, 10 repetition maximum; VO_2_peak, peak oxygen uptake; PAL, physical activity level. ^a^ Estimate of intervention effect at week 13 by using a mixed linear model including the baseline value in the outcome vector and adjusting for stratification factors (sex and SU-derivate use). ^b^ Analysis of body weight, body composition, and anthropometry was based on a modified ITT population, excluding a statistically influential outlier for leg muscle mass (see methods).

**Table 3:** Dietary intake in test and control group during intervention (including supplements).

	Test Group (*n* = 51)	Control Group (*n* = 54)	*p* Value ^a^
Energy intake, kcal/d	1804 ± 430	1731 ± 445	0.411
Protein, g/d	110 ± 23.2	77.0 ± 26.4	<0.001
Protein, g/kg BW/d	1.15 ± 0.31	0.82 ± 0.32	<0.001
Protein, % of energy	24.7 ± 3.7	17.8 ± 3.8	<0.001
Carbohydrate, % of energy	42.0 ± 5.7	46.9 ± 6.3	<0.001
Fat, % of energy	28.1 ± 6.1	30.9 ± 6.8	0.037
Saturated fat, % of energy	9.9 ± 2.8	11.7 ± 2.9	0.002
Mono-unsaturated fat, % of energy	9.7 ± 2.9	10.6 ± 3.1	0.148
Poly-unsaturated fat, % of energy	6.0 ± 2.0	6.0 ± 2.0	0.972

Data are presented as mean ± SD; intake data at week 13. BW, body weight. ^a^ Significance level (two-sided *p*-value) for comparison between groups using independent samples t-test.

## Data Availability

The data presented in this study are available on request from the corresponding author.

## Supplementary Materials

The following are available online at https://www.mdpi.com/2072-6643/13/1/64/s1, Table S1: Outcome measures for body weight, body composition, and anthropometry for test and control group with intervention effect, for ITT population; Table S2: Outcome measures for test and control group with intervention effect, excluding four subjects with pre-diabetes (sensitivity analysis); Figure S1: Identification of subject S094 as statistically influential outlier for leg muscle mass; Document S1: Exercise program PROBE study.

## Appendix A

**Table A1:** Composition of the study products, per serving of 150 mL.

Component	Test Product	Control Product
Energy (kcal)	150	150
Protein (g)	20.7	-
Leucine, total ^a^ (g)	2.8	-
EAA, total ^a^ (g)	10.6	-
Carbohydrates (g)	9.4	24.5
Sugars ^b^ (g)	4.2	15.6
Fat (g)	3.0	5.8
Saturated fat (g)	0.8	3.2
Mono-unsaturated fat (g)	1.7	2.1
Poly-unsaturated fat (g)	0.6	0.6
Fiber (g)	1.25	-
Vitamin D_3_^c^ (μg)	20	-
Calcium ^c^ (mg)	500	0.7

EAA, essential amino acids (Leu, Ile, Val, Phe, Met, His, Trp, Thr, and Lys); BCAA: branched-chain amino acids (Leu, Ile, and Val). ^a^ Provided by protein and free BCAA. ^b^ Consisting of lactose and fructose. ^c^ The test product also contained the following micronutrients: phosphorus (250 mg), magnesium (37 mg), iron (2.4 mg), zinc (2.2 mg), copper (270 mg), manganese (0.50 mg), fluoride (0.15 mg), molybdenum (15 μg), selenium (15 μg), chromium (7.5 μg), iodine (20 μg), vitamin A (152 μg retinol equivalents), vitamin E (7.5 mg α-tocopherol equivalents), vitamin K (12 μg), vitamin B-1 (0.23 mg), vitamin B-2 (0.25 mg), niacin (8.8 mg niacin equivalents), pantothenic acid (0.81 mg), vitamin B-6 (750 μg), folic acid (200 μg), vitamin B-12 (3.0 μg), biotin (6.1 μg), vitamin C (32 mg), carotenoids (300 μg), and choline (55 mg).
