# Supplementary material for: Effect of an Enriched Protein Drink on Muscle Mass and Glycemic Control during Combined Lifestyle Intervention in Older Adults with Obesity and Type 2 Diabetes: A Double-Blind RCT
Source: Nutrients. 2020 Dec 28;13(1):64. doi: 10.3390/nu13010064 (PMC7823734; doi:10.3390/nu13010064)
Supplement: Supplementary file 1 [file nutrients-13-00064-s001.zip › Document S1.pdf]

Document S1. Exercise program PROBE study

Introduction

The exercise program consisted of 3 supervised 1-hour group sessions per week, for 13 weeks. All exercise sessions were supervised by qualified personal trainers. The program consisted of progressive resistance exercise and high intensity interval training (HIIT).
Each training session was preceded by a 10-minute warming up on a cycle ergometer, cross trainer, or treadmill.

Resistance exercise

Resistance exercise involved a selection of 10 exercises focusing on the large muscle groups in legs and arms:
Legs:

- Leg press
- Leg curl
- Lunge
- Double knee raise
- Leg extension

Arms:

- Low row
- Chest press
- Arm curl
- Lateral pull down
- Fly

Subjects were familiarized to the exercises during the first week of intervention, in which subject's 1-RM (1 repetition maximum) was estimated for the specific exercise. After familiarization, exercise intensity started at 60% of subject's 1-RM (1st month), progressing to 70% (2nd month) and finally 80% (3rd month). Number of sets increased from 1 to 3 according to individual progress, involving 20 (at 60% 1-RM), 10-15 (70% 1-RM), and 8-10 (80% 1-RM) repetitions per set. The number of sets increased from 1 to 3 according to individual progress (exhaustion or failure after set 1, and after set 2: yes/no).

High Intensity Interval Training (HIIT)

After 2 weeks of training, subjects started HIIT on a cycle ergometer at 70% of their maximal work capacity, determined using a steep ramp test (see below). Intervals of 30 seconds of high-intensity exercise were followed by 60 seconds of active rest. Exercise intensity progressed to 90% (during the 2nd month), and finally 110% (during the 3rd month) of maximal work capacity. The number of intervals progressed from 4 to 8, according to individual progress [1].

Steep ramp test

Subjects underwent maximal cycle ergometry using a steep ramp test to determine their maximal work capacity and peak oxygen uptake. The steep ramp test has been validated against the common ramp test and was found to be a low-risk, accurate, and reliable test to measure exercise capacity in type 2 diabetes patients [2]. The steep ramp test starts with a 4 minute resting period followed by 2 minutes of unloaded cycling for warming-up. After warming-up, load increases linearly with 25 Watt every 10 seconds. The test stops at subject's exhaustion or giving up, or for safety reasons as judged by the assessor. Oxygen uptake and carbon dioxide exhalation is measured using breath-by-breath gas analysis (Quark RMR with CPET module, Cosmed). The highest average 10-second oxygen uptake is documented as the VO_2peak. The highest load completed is documented as Wmax.

1. Praet, S.F.; van Loon, L.J. Optimizing the therapeutic benefits of exercise in type 2 diabetes. *J Appl Physiol* **2007**, 103, 1113–1120.
2. Rozenberg, R.; Bussmann, J.B.; Lesaffre, E.; Stam, H.J.; Praet, S.F. A steep ramp test is valid for estimating maximal power and oxygen uptake during a standard ramp test in type 2 diabetes. *Scand J Med Sci Sports* **2015**, 25, 595-602.
