# Supplementary material for: Effect of an Enriched Protein Drink on Muscle Mass and Glycemic Control during Combined Lifestyle Intervention in Older Adults with Obesity and Type 2 Diabetes: A Double-Blind RCT
Source: Nutrients. 2020 Dec 28;13(1):64. doi: 10.3390/nu13010064 (PMC7823734; doi:10.3390/nu13010064)
Supplement: Supplementary file 1 [file nutrients-13-00064-s001.zip › Figure S1.pdf]

Figure S1. Identification of subject S094 as statistically influential outlier for leg muscle mass.

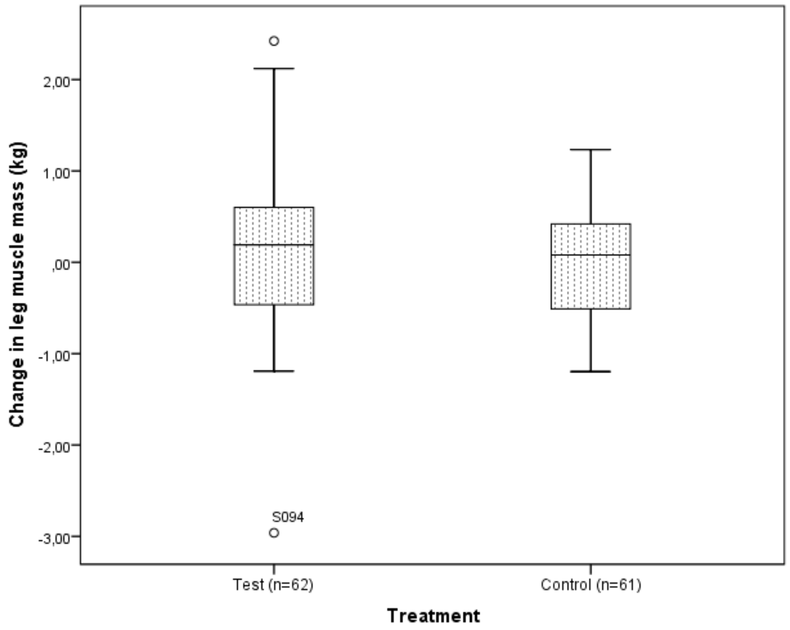

a) Boxplot for primary outcome parameter leg muscle mass, showing subject S094 as outlier. Whiskers indicate max/min observed value within 1.5*IQR from box. IQR: interquartile range.

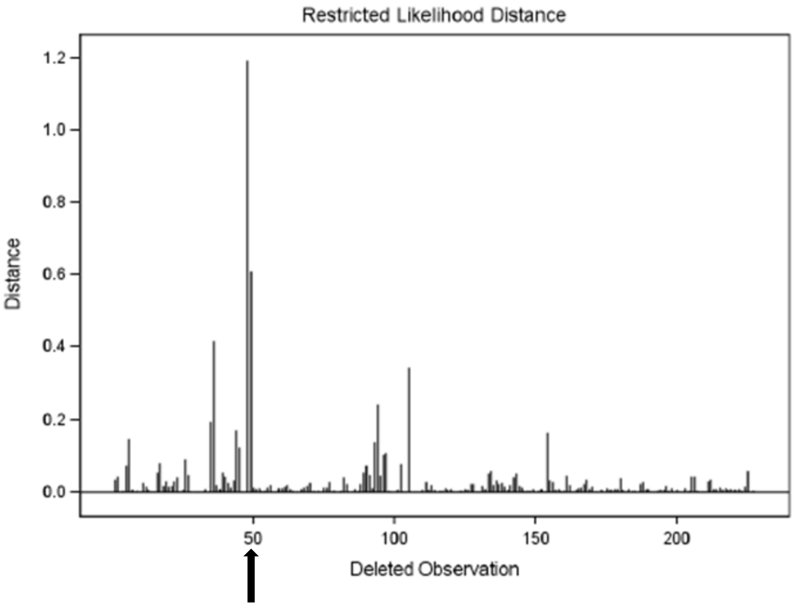

b) Restricted likelihood distance versus deleted observation, showing subject S094 as statistically influential outlier. This subject reported oedema in the legs at baseline and started on a sodium restricted diet during the study, which can be the explanation for the large reduction in lean mass as detected with DXA.
