# Supplementary material for: Effect of an Enriched Protein Drink on Muscle Mass and Glycemic Control during Combined Lifestyle Intervention in Older Adults with Obesity and Type 2 Diabetes: A Double-Blind RCT
Source: Nutrients. 2020 Dec 28;13(1):64. doi: 10.3390/nu13010064 (PMC7823734; doi:10.3390/nu13010064)
Supplement: Supplementary file 1 [file nutrients-13-00064-s001.zip › Table S1.pdf]

Table S1. Outcome measures for body weight, body composition, and anthropometry for test and control group with intervention effect, for ITT population.

		Test group	Control group	Intervention effect Beta (95% CI) [a]
Body weight, kg				
	Baseline (n)	96.57 ± 1.96 (62)	98.97 ± 1.92 (61)	
	Change (n)	-2.41 ± 0.43 (51)	-2.92 ± 0.41 (54)	0.50 (-0.67 to 1.68)
	P value	<0.001	<0.001	0.397
BMI, kg/m^2				
	Baseline (n)	33.1 ± 0.6 (62)	33.7 ± 0.6 (61)	
	Change (n)	-0.8 ± 0.1 (51)	-1.0 ± 0.1 (54)	0.2 (-0.2 to 0.6)
	P value	<0.001	<0.001	0.414
Leg muscle mass, kg				
	Baseline (n)	18.84 ± 0.36 (61)	19.21 ± 0.35 (61)	
	Change (n)	0.17 ± 0.11 (50)	-0.05 ± 0.11 (54)	0.21 (-0.10 to 0.52)
	P value	0.137	0.681	0.175
Appendicular muscle mass, kg				
	Baseline (n)	25.39 ± 0.48 (59)	25.92 ± 0.47 (61)	
	Change (n)	0.31 ± 0.14 (48)	0.03 ± 0.13 (51)	0.27 (-0.11 to 0.66)
	P value	0.029	0.811	0.158
Total lean mass, kg				
	Baseline (n)	60.73 ± 1.01 (60)	62.13 ± 0.99 (61)	
	Change (n)	0.42 ± 0.28 (49)	-0.35 ± 0.28 (52)	0.77 (-0.01 to 1.56)
	P value	0.137	0.208	0.053
Fat mass, kg				
	Baseline (n)	34.51 ± 1.16 (61)	35.26 ± 1.13 (61)	
	Change (n)	-2.68 ± 0.33 (50)	-2.60 ± 0.32 (52)	-0.08 (-1.00 to 0.84)
	P value	<0.001	<0.001	0.862
Waist circumference, cm				
	Baseline (n)	113.3 ± 1.4 (61)	115.1 ± 1.3 (60)	
	Change (n)	-3.5 ± 0.5 (50)	-3.7 ± 0.5 (52)	0.1 (-1.3 to 1.6)
	P value	<0.001	<0.001	0.864
VAT, cm^2				
	Baseline (n)	176.5 ± 7.0 (62)	181.0 ± 6.8 (61)	
	Change (n)	-19.2 ± 3.9 (51)	-17.3 ± 3.8 (54)	-1.9 (-12.7 to 8.9)
	P value	<0.001	<0.001	0.724

Data are presented as estimated marginal mean ± SE.
[a] Estimate of intervention effect at week 13 by using a mixed linear model including the baseline value in the outcome vector and adjusting for stratification factors (sex and SU-derivate use).
