# Supplementary material for: Effect of an Enriched Protein Drink on Muscle Mass and Glycemic Control during Combined Lifestyle Intervention in Older Adults with Obesity and Type 2 Diabetes: A Double-Blind RCT
Source: Nutrients. 2020 Dec 28;13(1):64. doi: 10.3390/nu13010064 (PMC7823734; doi:10.3390/nu13010064)
Supplement: Supplementary file 1 [file nutrients-13-00064-s001.zip › Table S2.pdf]

Table S2. Outcome measures for test and control group with intervention effect, excluding four subjects with pre-diabetes (sensitivity analysis).

		Test group	Control group	Intervention effect Beta (95% CI) [a]
Body weight [b], kg				
	Baseline (n)	96.74 ± 2.02 (59)	99.25 ± 1.93 (59)	
	Change (n)	-2.21 ± 0.42 (48)	-2.93 ± 0.41 (52)	0.72 (-0.44 to 1.89)
	P value	<0.001	<0.001	0.221
BMI [b], kg/m²				
	Baseline (n)	33.1 ± 0.6 (59)	33.8 ± 0.6 (59)	
	Change (n)	-0.7 ± 0.1 (48)	-1.0 ± 0.1 (52)	0.2 (-0.2 to 0.6)
	P value	<0.001	<0.001	0.236
Leg muscle mass [b], kg				
	Baseline (n)	18.76 ± 0.37 (58)	19.21 ± 0.35 (59)	
	Change (n)	0.24 ± 0.11 (47)	-0.08 ± 0.10 (52)	0.32 (0.03 to 0.62)
	P value	0.025	0.423	0.030
Appendicular muscle mass [b], kg				
	Baseline (n)	25.33 ± 0.49 (56)	25.91 ± 0.47 (59)	
	Change (n)	0.39 ± 0.13 (45)	-0.03 ± 0.12 (49)	0.42 (0.06 to 0.78)
	P value	0.003	0.835	0.021
Total lean mass [b], kg				
	Baseline (n)	60.74 ± 1.04 (57)	62.24 ± 1.00 (59)	
	Change (n)	0.57 ± 0.27 (46)	-0.46 ± 0.26 (50)	1.03 (0.29 to 1.77)
	P value	0.038	0.077	0.007
Fat mass [b], kg				
	Baseline (n)	34.62 ± 1.19 (58)	35.39 ± 1.14 (59)	
	Change (n)	-2.60 ± 0.34 (47)	-2.50 ± 0.33 (50)	-0.10 (-1.05 to 0.84)
	P value	<0.001	<0.001	0.830
Waist circumference [b], cm				
	Baseline (n)	113.5 ± 1.4 (58)	115.4 ± 1.3 (58)	
	Change (n)	-3.4 ± 0.5 (47)	-3.8 ± 0.5 (50)	0.4 (-1.1 to 1.8)
	P value	<0.001	<0.001	0.625
VAT [b], cm²				
	Baseline (n)	178.8 ± 7.3 (59)	182.1 ± 7.0 (59)	
	Change (n)	-18.7 ± 4.1 (48)	-17.1 ± 4.0 (52)	-1.6 (-12.9 to 9.7)
	P value	<0.001	<0.001	0.779
Fasting plasma glucose, mmol/L				
	Baseline (n)	8.45 ± 0.24 (55)	8.40 ± 0.23 (56)	
	Change (n)	-0.69 ± 0.24 (45)	-0.68 ± 0.23 (48)	0.02 (-0.68 to 0.64)
	P value	0.004	0.005	0.957
2h plasma glucose, mmol/L				
	Baseline (n)	16.00 ± 0.44 (55)	15.89 ± 0.42 (55)	
	Change (n)	-0.99 ± 0.37 (45)	-1.38 ± 0.37 (45)	0.39 (-0.66 to 1.44)
	P value	0.009	<0.001	0.465
HbA1c, mmol/mol				
	Baseline (n)	52.6 ± 1.2 (58)	54.1 ± 1.2 (56)	
	Change (n)	-4.4 ± 1.1 (47)	-5.8 ± 1.1 (49)	1.4 (-1.8 to 4.6)
	P value	<0.001	<0.001	0.384
Fasting plasma insulin, pmol/L				
	Baseline (n)	120.9 ± 8.1 (58)	104.9 ± 7.9 (57)	
	Change (n)	-20.6 ± 6.8 (46)	10.1 ± 6.7 (48)	-30.7 (-49.6 to -11.9)
	P value	0.003	0.132	0.002
HOMA-IR				
	Baseline (n)	6.43 ± 0.46 (55)	5.57 ± 0.45 (55)	
	Change (n)	-1.44 ± 0.42 (44)	0.14 ± 0.41 (47)	-1.58 (-2.76 to -0.41)
	P value	0.001	0.730	0.009

Matsuda index				
	Baseline (n)	2.12 ± 0.18 (53)	2.20 ± 0.18 (53)	
	Change (n)	0.52 ± 0.17 (41)	-0.01 ± 0.17 (42)	0.53 (0.07 to 1.00)
	P value	0.002	0.941	0.025
Serum calcidiol, nmol/L				
	Baseline (n)	64.4 ± 3.0 (55)	61.7 ± 2.9 (56)	
	Change (n)	17.8 ± 2.8 (43)	-4.1 ± 2.7 (46)	21.9 (14.2 to 29.7)
	P value	<0.001	0.134	<0.001
10-RM leg press, kg				
	Baseline (n)	125 ± 9 (53)	119 ± 8 (52)	
	Change (n)	49 ± 7 (34)	56 ± 7 (39)	-7 (-27 to 13)
	P value	<0.001	<0.001	0.484
Knee extension power, Watt				
	Baseline (n)	335 ± 17 (48)	341 ± 16 (51)	
	Change (n)	30 ± 8 (34)	35 ± 8 (38)	-5 (-27 to 18)
	P value	<0.001	<0.001	0.677
400-m walk speed, m/s				
	Baseline (n)	1.36 ± 0.03 (59)	1.45 ± 0.03 (56)	
	Change (n)	0.07 ± 0.02 (46)	0.03 ± 0.02 (49)	0.04 (-0.01 to 0.09)
	P value	<0.001	0.064	0.139
Usual gait speed, m/s				
	Baseline (n)	1.11 ± 0.03 (60)	1.17 ± 0.03 (58)	
	Change (n)	0.01 ± 0.03 (48)	-0.03 ± 0.03 (51)	0.04 (-0.04 to 0.11)
	P value	0.830	0.283	0.366
Chair stand, s				
	Baseline (n)	12.2 ± 0.3 (60)	11.8 ± 0.3 (56)	
	Change (n)	-1.4 ± 0.3 (48)	-1.3 ± 0.3 (48)	-0.1 (-0.9 to 0.7)
	P value	<0.001	<0.001	0.772
VO_2peak, l/min				
	Baseline (n)	1.60 ± 0.06 (59)	1.75 ± 0.05 (58)	
	Change (n)	0.13 ± 0.04 (40)	0.11 ± 0.04 (46)	0.03 (-0.08 to 0.13)
	P value	0.001	0.003	0.615
PAL				
	Baseline (n)	1.18 ± 0.01 (51)	1.19 ± 0.01 (55)	
	Change (n)	0.01 ± 0.01 (40)	0.00 ± 0.01 (43)	0.01 (-0.02 to 0.04)
	P value	0.259	0.714	0.576

Data are presented as estimated marginal mean ± SE.

[a] Estimate of intervention effect at week 13 by using a mixed linear model including the baseline value in the outcome vector and adjusting for stratification factors (sex and SU-derivate use).

[b] Analysis of body weight, body composition, and anthropometry was based on a modified ITT population, excluding a statistically influential outlier for leg muscle mass (see methods).
